# Physical activity and the risk of periodontitis: an instrumental variable study

**DOI:** 10.1007/s00784-023-05109-9

**Published:** 2023-06-13

**Authors:** Sebastian-Edgar Baumeister, Stefan Lars Reckelkamm, Benjamin Ehmke, Michael Nolde, Hansjörg Baurecht

**Affiliations:** 1 Institute of Health Services Research in Dentistry, University of Münster, Albert-Schweitzer-Campus 1, 48149 Münster, Germany; 2 Clinic for Periodontology and Conservative Dentistry, University of Münster, Albert-Schweitzer-Campus 1, 48149 Münster, Germany; 3 Department of Epidemiology and Preventive Medicine, University of Regensburg, Franz-Josef-Strauss-Allee 11, 93053 Regensburg, Germany

**Keywords:** Periodontitis, Physical activity, Etiology, Prevention

## Abstract

**Objectives:**

Observational studies suggested an inverse association between physical activity and periodontitis. However, observational studies might be subject to unobserved confounding and reverse causation bias. We conducted an instrumental variable study to strengthen the evidence on the relationship between physical activity and periodontitis.

**Materials and methods:**

We used genetic variants associated with self-reported and accelerometer-assessed physical activity in 377,234 and 91,084 UK Biobank participants, respectively, as instruments. For these instruments, genetic associations with periodontitis were obtained from 17,353 cases and 28,210 controls in the GeneLifestyle Interactions in Dental Endpoints consortium.

**Results:**

We found no evidence for effects of self-reported moderate-to-vigorous physical activity, self-reported vigorous physical activity, accelerometry “average accelerations,” and “fraction of accelerations > 425 milli-gravities” on periodontitis. For example, the odds ratio for self-reported moderate-to-vigorous physical activity was 1.07 (95% credible interval: 0.87; 1.34) in Causal Analysis using Summary Effect Estimates. We conducted sensitivity analyses to rule out weak instrument bias and correlated horizontal pleiotropy.

**Conclusions:**

The study does not support an effect of physical activity on the risk of periodontitis.

**Clinical relevance:**

This study provides little evidence that recommending physical activity would help prevent periodontitis.

**Supplementary Information:**

The online version contains supplementary material available at 10.1007/s00784-023-05109-9.

## Introduction

Metabolic conditions, including obesity and diabetes, and inflammatory conditions such as rheumatic diseases are linked to periodontitis [1, 2]. Physical inactivity is considered a modifiable risk factor for metabolic and inflammatory disease development and progression [3, 4]. Meta-analysis of observational data strongly suggests that physical activity is prospectively related to a lower incidence of diabetes [5]. Regular physical activity can reduce diabetes risk by enhancing energy expenditure, insulin action through increased muscle glucose uptake, and muscle insulin sensitivity [6]. The observational literature further shows that physical activity is associated with reduced risk of rheumatoid arthritis [7]. Modulation of inflammation is one of the postulated mechanisms behind the apparent relationship between physical activity and rheumatic arthritis [6]. Genetically instrumented physical activity was further found to be associated with fewer lymphocytes and eosinophils, suggesting a potential effect of physical activity in improving the inflammatory state [8].

Several studies have also suggested an inverse association between physical activity and periodontitis [9, 10]. However, the available studies on physical activity and periodontitis are observational. Observational studies are vulnerable to various biases such as confounding (low physical activity may correlate with other periodontitis risk factors) or reverse causation (symptoms of periodontitis may result in reduction of physical activity). Confounding by measured factors can be adjusted for using approaches such as multivariable regression, propensity score matching, and inverse probability weighting [11]. The validity of the estimates obtained from such methods relies on the assumption that all confounders have been measured and adjusted for. These potential biases may limit the validity of findings derived from traditional observational research [12]. Instrumental variable analysis is an approach to obtain unbiased inference even in the presence of unobserved confounders and reverse causation [13, 14]. We employed genetic variants from genome-wide association studies (GWAS) of physical activity traits as instruments to examine the effect of self-reported and accelerometer-assessed physical activity on the risk of periodontitis in a two-sample instrumental variable study.

## Materials and methods

### Genotype data for physical activity

We used GWAS-level genotyping data from a GWAS of self-reported and accelerometer-based physical activity conducted in the UK Biobank [15] (Table 1). UK Biobank is a community-based prospective cohort study that recruited over 500,000 men and women ages 40–69 [16]. Self-reported levels of physical activity were ascertained in 377,234 UK Biobank participants using the International Physical Activity Questionnaire Short Form [17]. Moderate-to-vigorous physical activity (MVPA) was computed by taking the sum of total minutes per week of moderate and vigorous physical activity multiplied by eight, corresponding to their metabolic equivalents [15]. Individuals who met 3 or more days per week of vigorous physical activity (VPA) were classified as vigorously physical active [15].

**Table 1:** Genome-wide association studies used to obtain genetic associations for physical activity and periodontitis

Phenotype	Sample size	Cases	Controls	First author (year, PMID)
Self-reported moderate-to-vigorous physical activity	377,234			Klimentidis (2018, 29899525)
Self-reported vigorous physical activity		98,060	162,995	Klimentidis (2018, 29899525)
Accelerometery—average acceleration	91,084			Klimentidis (2018, 29899525)
Accelerometery—fraction of time with acceleration 425 milli-gravities	90,667			Klimentidis (2018, 29899525)
Periodontitis	45,563	17353	28210	Shungin (2019, 31235808)

*PMID* PubMed identifier

For objective assessment of physical activity, a subset of 103,712 participants wore an Axivity AX3 triaxial accelerometer on the wrist for a 7-day period between 2013 and 2015 [18]. After calibration, removal of gravity and sensor noise, and identification of wear/nonwear episodes, the remaining 100 Hz raw triaxial acceleration data was used to calculate physical activity variables. For the GWAS [15], “average acceleration” (AVEACC) (in milli-gravities (mg)) was used as the exposure variable derived from accelerometer wear. GWAS of fraction of accelerations > 425 mg (ACC425) was also derived because it corresponds to an equivalent of VPA (six metabolic equivalent of tasks (MET)) [19].

### Genetic associations with periodontitis

Genetic association estimates for periodontitis of instruments for physical activity traits were obtained from a GWAS of European studies contributing to the GeneLifestyle Interactions in Dental Endpoints (GLIDE) consortium, totaling 17,353 clinical periodontitis cases and 28,210 controls (Table 1) [20]. Periodontitis cases were classified by either the Centers for Disease Control and Prevention/American Academy of Periodontology or the Community Periodontal Index case definition.

### Power calculation

Power calculations were performed separately for each physical activity-periodontitis combination in inverse variance weighted (IVW) analyses (see “Statistical analysis”) according to [21]. Given *α* = 5%, we had ≥ 80% power when the expected odds ratios (OR) for periodontitis were ≤ 0.91, ≤ 0.89, ≤ 0.87, ≤ 0.80 for MVPA, VPA, AVEACC, and ACC425, respectively.

### Statistical analysis

We used two-sample instrumental variable estimation based on GWAS summary statistics to estimate effects of genetically instrumented physical activity exposures on periodontitis risk. Causal Analysis using Summary Effect Estimates (CAUSE) [22] was used as principal analytic device because it maximizes power (relative to other instrumental variable methods) by utilizing large sets of single nucleotide polymorphisms (SNPs) and its robustness to weak instrument bias and correlated horizontal pleiotropy [14, 22]. It uses genome-wide summary statistics to disentangle causality (i.e., SNPs are associated with periodontitis through their effect on physical activity) from correlated horizontal pleiotropy (i.e., SNPs are associated with physical activity and periodontitis through a shared heritable factor), while taking into account uncorrelated horizontal pleiotropy (i.e., SNPs are associated with physical activity through separate mechanisms). It uses Bayesian modelling to assess whether the sharing model (i.e., model that fixes the causal effect at zero) fits the data at least as well as the causal model (i.e., model that allows a causal effect different from zero).

For secondary analysis, we applied IVW analysis for weak, correlated instruments [23], and the robust adjusted profile score (RAPS) methodology [24], which enables the use of weak instruments, is robust to horizontal pleiotropy and considers potential measurement error in the exposure estimate. For IVW and RAPS, we used the clump_data function in the TwoSampleMR R package to select SNPs (*P* < 5 × 10^−6^) for each physical activity measure and performed linkage disequilibrium (LD) clumping (r^2^ < 0.2) using the European reference panel from the 1000 Genomes Project Phase 3 v5 (Supplementary Table 1). For SNPs not found in the outcome datasets, we used proxy SNP with a high LD (r^2^ > 0.9). Results are presented as odds ratio (OR) per 1-standard deviation increment in MVPA (MET-minutes/week) or AVEACC. One standard deviation of AVEACC in the UK Biobank Study is approximately 8 milli-gravities (or 0.08 m/s^2^) of acceleration in a mean 5-s window [15]. The OR estimates for VPA were scaled to a doubling in prevalence [25]. A Benjamini–Hochberg false discovery rate (FDR) < 5% was used to correct the results for multiple testing.

Instrumental variable estimation assumes instruments are (1) associated with physical activity (relevance assumption), (2) independent of confounders of the association between the instruments and periodontitis (exchangeability assumption), and (3) only associated with periodontitis through their effect on physical activity (exclusion restriction assumption) [14, 26]. To test for potential violations of the relevance assumption, we calculated F-statistics and the R^2^ for each measure of physical activity. Violations of the exchangeability and exclusion restriction assumption can occur when the instrument affects the outcome but through different pathways. Although it is not possible to prove that the exchangeability and exclusion restriction assumptions hold, sensitivity analysis can be used to uncover possible violations of these assumptions. One approach includes the assessment of potential violation of the exclusion restriction assumption through evaluation of the heterogeneity of the individual SNP estimates and through pleiotropy-robust approaches, such as CAUSE or RAPS, which provide unbiased estimates in the presence of horizontal pleiotropy [14]. We assessed heterogeneity using Cochran *Q* and *I*_GX_^2^ statistics. The MR Egger intercept test was used to test for directional pleiotropy [26]. We performed the analysis using R version 4.1.3 (R Foundation for Statistical Computing) using the cause, MendelianRandomization, mr.raps, and TwoSampleMR packages. The study was conducted based on relevant analysis and reporting guidelines [27, 28].

## Results

In our main analysis, genetically instrumented physical activity was analyzed in relation to periodontitis using Causal Analysis using Summary Effect Estimates (CAUSE) [22]. We did not find evidence supporting effects of moderate-to-vigorous physical activity (MVPA), vigorous physical activity (VPA), average acceleration (AVEACC), or fraction of accelerations > 425 mg (ACC425) on the risk of periodontitis (Table 2). For example, the OR for MVPA and AVEACC were 1.07 (95% credible interval: 0.87; 1.34) and 1.00 (95% credible interval: 0.98; 1.02), respectively. The CAUSE estimates were supported IVW and RAPS analyses (Table 2).

**Table 2:** Effect of genetically instrumented physical activity traits on periodontitis

Exposure	Method	OR	95% CI	*P*-value^1^	FDR
Self-reported moderate-to-vigorous physical activity	CAUSE	1.07	0.87; 1.34	1.000	-
	IVW	0.97	0.78; 1.21	0.7840	0.8043
	RAPS	0.97	0.76; 1.25	0.8404	0.8887
Self-reported vigorous physical activity	CAUSE	0.90	0.50; 1.77	0.944	-
	IVW	1.07	0.64; 1.78	0.8043	0.8043
	RAPS	1.04	0.62; 1.75	0.8887	0.8887
Accelerometery—average acceleration	CAUSE	1.00	0.98; 1.02	1.000	-
	IVW	1.01	0.99; 1.03	0.4551	0.8043
	RAPS	1.02	0.99; 1.04	0.1277	0.5108
Accelerometery—fraction of time with acceleration 425 milli-gravities	CAUSE	0.99	0.81; 1.23	1.000	-
	IVW	0.93	0.70; 1.23	0.5951	0.8043
	RAPS	0.89	0.64; 1.23	0.4709	0.8887

*CAUSE* Causal analysis using summary effect estimates, *CI* confidence or credible (of CAUSE) interval, *FDR* false discovery rate adjusted *P*-value, *IVW* inverse variance-weighted model, *OR* odds ratio per standard deviation increment in physical activity exposure or doubling of “self-reported vigorous physical activity,” *RAPS* robust-adjusted profile score

^1^CAUSE: *P*-value of Bayesian model comparison

The instruments for MVPA, VPA, AVEACC, or ACC425 explained 8.8%, 5.8%, 3.8%, and 1.5% of the phenotypic variance. The minimum F-statistic was 20.9, suggesting sufficient instrument strength and no violation of the relevance assumption (Supplementary Table 1). There was no heterogeneity in the IVW analyses (Supplementary Table 2). The intercepts from the MR Egger regression were centered around zero and provided no evidence for unbalanced pleiotropy (Supplementary Table 2).

## Discussion

This instrumental variable study used genetic variants as instruments for self-reported and accelerometer-assessed physical activity to estimate effects on periodontitis risk. The findings do not suggest effects of physical activity on periodontitis. The study leveraged the CAUSE approach that maximizes statistical power and is robust to weak instrument bias and correlated horizontal pleiotropy. The CAUSE findings were endorsed in sensitivity analyses that aimed to further rule out that weak instrument bias or violations of the exchangeability or exclusion restriction instrumental variable assumptions are responsible for the null findings.

The instrumental variable approach using genetic variants as instruments has been successfully applied to shed light on the relationships of physical activity with various cancers [29, 30], cardiovascular diseases [31], psychiatric diseases [32], and neurological conditions [33]. In part, these instrumental variable studies refuted the widely held view that exercise medicine is a universal tool for disease prevention. One example is dementia, where a large number of observational cohort studies showed an inverse association of physical activity and disease risk. Subsequent long-term cohort and instrumental variable studies revealed that the observed inverse association between physical activity and dementia was subject to reverse causation due to a decline in physical activity in prodromal disease [33, 34].

All available observational studies, except one [35], on physical activity and periodontitis are cross-sectional reducing the potential to infer cause-effect relations because the cross-sectional design does not ensure that the exposure precedes the outcome. With cross-sectional data, it is difficult to rule out reverse causation. In such designs, periodontal disease features may make individuals become less physically active and induce a spurious inverse association [36]. Another potential source of bias of the existing observational studies is unobserved confounding. For example, in a recent cross-sectional study [10], the authors used multivariable logistic regression to adjust the relationship between self-reported physical activity and periodontitis for age, sex, race, poverty level, education, smoking, and diabetes. Yet, there might have been additional unadjusted confounders (e.g., periodontitis risk factors that co-occur with low physical activity such as alcohol consumption, oral hygiene, regular dental visits) or partially adjusted confounders (e.g., due to imperfect measurement of smoking or non-linear association with age) that might have introduced residual confounding.

The study has limitations. First, the study population comprised only individuals of European ancestry. Although restricting the study to ethnically homogeneous populations minimizes population-stratification bias, our results may not be generalizable to other populations with different genetic backgrounds. Second, genetic variants for ACC425 only explained 1.5% of the phenotypic variability, which may have reduced statistical power. In the future, identification of more instruments that explain more variance in accelerometer-assessed vigorous activity could strengthen inference. Third, the GWAS of physical activity consisted of UK Biobank participants aged 40 to 70 years. Previous twin studies [37] suggest that the genetic contribution to physical activity decreases with age. By estimating instrument-physical activity associations in a sample of middle-aged and older adults, we might have underestimated the denominator effect of the ratio estimator [33]. Thus, when the effect of the instrument on exposure changes over time, the ratio estimator could represent a biased estimate of the effect of physical activity on periodontitis.

In conclusion, the present study provides little evidence that physical activity would help to prevent periodontitis. Large cohort studies with repeated and valid measurement of physical activity and periodontitis and large GWAS data to provide strong instruments for physical activity in instrumental variable studies are needed to further triangulate the available evidence [12].

## Supplementary Information

Below is the link to the electronic supplementary material.

Supplementary file1 (DOCX 140 KB)

## Data Availability

All data analyzed for this study are publicly available. The UK Biobank physical activity data can be accessed at https://klimentidis.lab.arizona.edu/content/data. Periodontitis summary data are available at https://data.bris.ac.uk/data/dataset/2j2rqgzedxlq02oqbb4vmycnc2.
