# Supplementary material for: Physical activity and the risk of periodontitis: an instrumental variable study
Source: Clin Oral Investig. 2023 Jun 13;27(8):4803–8. doi: 10.1007/s00784-023-05109-9 (PMC10415453; doi:10.1007/s00784-023-05109-9)
Supplement: Supplementary file 1 — Supplementary file1 (DOCX 140 KB) [file 784_2023_5109_MOESM1_ESM.docx]

# Physical activity and the risk of periodontitis: An instrumental variable study

Sebastian-Edgar Baumeister et al.

Supplementary Tables

Supplementary Table 1 Associations of single nucleotide polymorphisms for physical activity traits used in inverse variance weighted and robust adjusted profile score analyses

|  |  |  |  | Estimates for physical activity | | | | Estimates for periodontitis | | |
| --- | --- | --- | --- | --- | --- | --- | --- | --- | --- | --- |
| SNP | EA | OA | EAF | BETA | SE | P | F | BETA | SE | P |
| Self-reported moderate-to-vigorous physical activity | | | | | | | | | | |
| rs10041140 | A | T | 0.733 | 0.012 | 0.003 | 7.900e-07 | 24.4 | 0.027 | 0.018 | 0.126 |
| rs10145335 | G | A | 0.749 | -0.014 | 0.003 | 2.700e-08 | 30.9 | -0.016 | 0.018 | 0.367 |
| rs10157145 | T | C | 0.496 | -0.012 | 0.002 | 9.600e-08 | 28.5 | -0.016 | 0.016 | 0.314 |
| rs10160758 | T | C | 0.733 | 0.013 | 0.003 | 1.700e-07 | 27.4 | -0.017 | 0.018 | 0.344 |
| rs1043595 | G | A | 0.717 | 0.014 | 0.002 | 4.300e-09 | 34.5 | -0.002 | 0.018 | 0.920 |
| rs1045958 | C | T | 0.660 | 0.011 | 0.002 | 2.600e-06 | 22.1 | -0.012 | 0.016 | 0.482 |
| rs10506671 | G | A | 0.911 | -0.019 | 0.004 | 6.800e-07 | 24.7 | -0.021 | 0.023 | 0.356 |
| rs1053924 | T | C | 0.314 | 0.012 | 0.002 | 8.800e-07 | 24.2 | -0.032 | 0.019 | 0.089 |
| rs10835224 | T | G | 0.690 | -0.011 | 0.002 | 4.100e-06 | 21.2 | -0.008 | 0.017 | 0.640 |
| rs11048486 | C | A | 0.835 | 0.014 | 0.003 | 3.400e-06 | 21.6 | -0.022 | 0.021 | 0.302 |
| rs11081859 | G | A | 0.108 | 0.018 | 0.004 | 2.100e-07 | 26.9 | 0.017 | 0.021 | 0.434 |
| rs111721461 | T | A | 0.933 | 0.021 | 0.004 | 1.200e-06 | 23.6 | 0.081 | 0.043 | 0.060 |
| rs111823604 | A | C | 0.708 | 0.013 | 0.002 | 1.800e-07 | 27.2 | 0.020 | 0.017 | 0.221 |
| rs112016448 | C | T | 0.991 | -0.062 | 0.013 | 2.300e-06 | 22.3 | 0.118 | 0.067 | 0.080 |
| rs112308536 | A | G | 0.679 | 0.011 | 0.002 | 4.900e-06 | 20.9 | 0.007 | 0.017 | 0.696 |
| rs11240359 | G | A | 0.822 | -0.015 | 0.003 | 8.700e-08 | 28.6 | -0.030 | 0.019 | 0.124 |
| rs114243593 | A | G | 0.947 | 0.027 | 0.005 | 5.500e-08 | 29.5 | 0.009 | 0.054 | 0.872 |
| rs114524724 | C | G | 0.975 | 0.035 | 0.007 | 9.000e-07 | 24.1 | 0.038 | 0.062 | 0.541 |
| rs115181288 | C | T | 0.945 | -0.023 | 0.005 | 2.300e-06 | 22.3 | 0.053 | 0.043 | 0.218 |
| rs11586691 | T | C | 0.502 | 0.011 | 0.002 | 5.800e-07 | 25.0 | 0.010 | 0.015 | 0.518 |
| rs11605988 | A | T | 0.723 | 0.013 | 0.002 | 1.200e-07 | 28.1 | -0.035 | 0.018 | 0.047 |
| rs11616749 | G | A | 0.783 | -0.013 | 0.003 | 2.900e-06 | 21.9 | 0.016 | 0.022 | 0.480 |
| rs116368148 | C | G | 0.960 | -0.026 | 0.006 | 4.200e-06 | 21.2 | 0.079 | 0.045 | 0.078 |
| rs11641100 | C | T | 0.556 | 0.012 | 0.002 | 1.600e-07 | 27.4 | 0.015 | 0.016 | 0.351 |
| rs117303800 | A | T | 0.778 | -0.012 | 0.003 | 3.700e-06 | 21.4 | -0.022 | 0.022 | 0.302 |
| rs117467952 | C | T | 0.910 | -0.019 | 0.004 | 5.800e-07 | 25.0 | -0.014 | 0.032 | 0.672 |
| rs11772444 | G | A | 0.811 | -0.019 | 0.003 | 4.700e-11 | 43.3 | 0.014 | 0.021 | 0.494 |
| rs11823140 | G | T | 0.703 | 0.011 | 0.002 | 3.000e-06 | 21.8 | -0.003 | 0.017 | 0.875 |
| rs1186721 | G | A | 0.684 | -0.013 | 0.002 | 4.400e-08 | 30.0 | 0.006 | 0.018 | 0.734 |
| rs11913445 | C | A | 0.832 | 0.015 | 0.003 | 3.900e-07 | 25.8 | -0.006 | 0.023 | 0.776 |
| rs12142550 | C | T | 0.750 | 0.013 | 0.003 | 1.300e-07 | 27.9 | 0.020 | 0.019 | 0.283 |
| rs12233587 | C | G | 0.717 | -0.012 | 0.002 | 2.600e-06 | 22.1 | -0.007 | 0.017 | 0.674 |
| rs12360330 | C | T | 0.865 | -0.016 | 0.003 | 1.300e-06 | 23.4 | -0.001 | 0.022 | 0.963 |
| rs12730457 | C | A | 0.516 | 0.011 | 0.002 | 3.000e-07 | 26.3 | 0.006 | 0.016 | 0.689 |
| rs12912808 | C | T | 0.851 | 0.018 | 0.003 | 1.700e-08 | 31.9 | -0.004 | 0.023 | 0.870 |
| rs12979056 | G | A | 0.542 | -0.010 | 0.002 | 2.300e-06 | 22.3 | -0.020 | 0.016 | 0.216 |
| rs1340704 | C | T | 0.402 | 0.010 | 0.002 | 4.700e-06 | 21.0 | -0.004 | 0.016 | 0.818 |
| rs139907649 | G | A | 0.991 | -0.059 | 0.012 | 1.500e-06 | 23.1 | -0.198 | 0.362 | 0.585 |
| rs144143828 | C | T | 0.908 | 0.019 | 0.004 | 4.400e-07 | 25.5 | 0.015 | 0.031 | 0.628 |
| rs1469851 | A | T | 0.198 | 0.014 | 0.003 | 1.000e-06 | 23.9 | 0.039 | 0.019 | 0.040 |
| rs149432883 | G | A | 0.891 | 0.017 | 0.004 | 8.700e-07 | 24.2 | 0.014 | 0.030 | 0.658 |
| rs150863806 | G | A | 0.969 | 0.032 | 0.007 | 1.300e-06 | 23.4 | -0.018 | 0.066 | 0.778 |
| rs161344 | C | A | 0.568 | -0.010 | 0.002 | 4.000e-06 | 21.3 | -0.003 | 0.016 | 0.843 |
| rs16897515 | C | A | 0.810 | 0.015 | 0.003 | 5.300e-08 | 29.6 | -0.018 | 0.021 | 0.383 |
| rs17183317 | A | G | 0.937 | -0.022 | 0.005 | 2.100e-06 | 22.5 | 0.042 | 0.034 | 0.216 |
| rs17573850 | A | G | 0.741 | 0.012 | 0.003 | 1.700e-06 | 22.9 | 0.022 | 0.019 | 0.234 |
| rs1793890 | G | A | 0.674 | 0.012 | 0.002 | 2.000e-07 | 27.1 | 0.021 | 0.024 | 0.386 |
| rs184749094 | C | T | 0.988 | -0.052 | 0.011 | 3.700e-06 | 21.4 | 0.170 | 0.246 | 0.488 |
| rs1873585 | A | G | 0.778 | -0.012 | 0.003 | 4.400e-06 | 21.1 | 0.003 | 0.018 | 0.876 |
| rs189635394 | G | A | 0.968 | 0.030 | 0.006 | 3.300e-06 | 21.6 | -0.046 | 0.059 | 0.426 |
| rs1921981 | G | A | 0.674 | 0.013 | 0.002 | 3.800e-08 | 30.2 | 0.017 | 0.017 | 0.316 |
| rs1955896 | A | G | 0.538 | -0.010 | 0.002 | 3.800e-06 | 21.4 | 0.015 | 0.016 | 0.351 |
| rs1972763 | C | T | 0.342 | 0.013 | 0.002 | 3.300e-08 | 30.5 | 0.017 | 0.016 | 0.309 |
| rs1974771 | G | A | 0.900 | -0.021 | 0.004 | 6.600e-09 | 33.7 | -0.003 | 0.025 | 0.917 |
| rs2035562 | A | G | 0.328 | -0.014 | 0.002 | 3.900e-09 | 34.7 | -0.004 | 0.017 | 0.827 |
| rs204991 | T | C | 0.780 | 0.014 | 0.003 | 1.100e-07 | 28.3 | -0.013 | 0.024 | 0.590 |
| rs2050189 | T | C | 0.771 | 0.012 | 0.003 | 3.500e-06 | 21.5 | -0.012 | 0.026 | 0.654 |
| rs2073045 | G | A | 0.626 | 0.011 | 0.002 | 2.400e-06 | 22.2 | 0.001 | 0.023 | 0.977 |
| rs2114286 | A | G | 0.466 | -0.012 | 0.002 | 3.300e-08 | 30.5 | 0.002 | 0.016 | 0.891 |
| rs2148201 | G | A | 0.509 | -0.011 | 0.002 | 1.700e-06 | 22.9 | 0.008 | 0.016 | 0.596 |
| rs2267443 | A | G | 0.381 | 0.011 | 0.002 | 3.400e-06 | 21.6 | -0.034 | 0.016 | 0.041 |
| rs2267549 | G | A | 0.546 | 0.011 | 0.002 | 2.800e-06 | 22.0 | -0.020 | 0.016 | 0.198 |
| rs2283249 | T | G | 0.752 | -0.013 | 0.003 | 1.100e-06 | 23.8 | 0.004 | 0.018 | 0.840 |
| rs2388195 | A | C | 0.561 | -0.012 | 0.002 | 1.700e-07 | 27.4 | 0.013 | 0.016 | 0.403 |
| rs2413357 | T | A | 0.277 | 0.012 | 0.002 | 1.900e-06 | 22.7 | 0.007 | 0.017 | 0.674 |
| rs2451292 | T | G | 0.473 | -0.010 | 0.002 | 4.000e-06 | 21.3 | 0.010 | 0.016 | 0.534 |
| rs2494664 | A | G | 0.540 | -0.012 | 0.002 | 8.900e-08 | 28.6 | -0.022 | 0.016 | 0.166 |
| rs2638463 | G | A | 0.694 | -0.012 | 0.003 | 4.600e-06 | 21.0 | -0.002 | 0.017 | 0.888 |
| rs2705570 | A | G | 0.294 | 0.012 | 0.002 | 1.300e-06 | 23.4 | 0.020 | 0.017 | 0.238 |
| rs2710477 | T | A | 0.919 | -0.021 | 0.004 | 3.500e-07 | 25.9 | 0.001 | 0.026 | 0.963 |
| rs2721937 | C | T | 0.815 | -0.014 | 0.003 | 1.400e-06 | 23.3 | 0.040 | 0.019 | 0.039 |
| rs2764261 | A | G | 0.374 | 0.011 | 0.002 | 6.300e-07 | 24.8 | 0.003 | 0.016 | 0.867 |
| rs2854277 | C | T | 0.917 | 0.032 | 0.005 | 2.600e-10 | 40.0 | -0.049 | 0.040 | 0.220 |
| rs2905724 | G | A | 0.678 | 0.012 | 0.002 | 3.900e-07 | 25.7 | 0.003 | 0.019 | 0.868 |
| rs293566 | T | C | 0.669 | -0.011 | 0.002 | 2.600e-06 | 22.1 | -0.021 | 0.017 | 0.208 |
| rs2942127 | G | A | 0.175 | 0.016 | 0.003 | 3.300e-08 | 30.5 | -0.005 | 0.020 | 0.804 |
| rs2988004 | T | G | 0.558 | -0.013 | 0.002 | 4.100e-09 | 34.6 | 0.020 | 0.016 | 0.209 |
| rs3094622 | A | G | 0.865 | 0.020 | 0.003 | 1.400e-09 | 36.6 | -0.008 | 0.040 | 0.842 |
| rs3095303 | G | A | 0.682 | 0.011 | 0.002 | 4.800e-06 | 20.9 | -0.014 | 0.017 | 0.423 |
| rs3117110 | C | T | 0.784 | 0.014 | 0.003 | 8.800e-08 | 28.6 | -0.021 | 0.026 | 0.418 |
| rs34467919 | T | C | 0.381 | 0.010 | 0.002 | 4.200e-06 | 21.2 | -0.016 | 0.016 | 0.318 |
| rs35650232 | T | G | 0.779 | -0.013 | 0.003 | 2.500e-06 | 22.1 | 0.015 | 0.022 | 0.501 |
| rs3810496 | T | C | 0.384 | 0.011 | 0.002 | 1.500e-06 | 23.1 | -0.013 | 0.017 | 0.452 |
| rs3936938 | G | A | 0.403 | -0.011 | 0.002 | 1.600e-06 | 23.0 | 0.005 | 0.016 | 0.753 |
| rs4281659 | T | C | 0.865 | 0.017 | 0.003 | 1.000e-07 | 28.3 | -0.029 | 0.021 | 0.178 |
| rs429358 | T | C | 0.846 | -0.022 | 0.003 | 6.100e-13 | 51.8 | 0.011 | 0.023 | 0.622 |
| rs4304660 | G | A | 0.273 | -0.011 | 0.002 | 4.500e-06 | 21.0 | -0.003 | 0.018 | 0.854 |
| rs466727 | G | C | 0.329 | 0.011 | 0.002 | 3.100e-06 | 21.7 | -0.002 | 0.017 | 0.907 |
| rs469228 | A | G | 0.748 | 0.013 | 0.003 | 3.900e-07 | 25.8 | -0.003 | 0.018 | 0.869 |
| rs4787924 | G | A | 0.475 | -0.011 | 0.002 | 8.900e-07 | 24.2 | 0.007 | 0.015 | 0.652 |
| rs4800990 | C | T | 0.434 | -0.011 | 0.002 | 1.900e-06 | 22.7 | 0.021 | 0.016 | 0.173 |
| rs4821271 | G | C | 0.218 | 0.013 | 0.003 | 5.100e-07 | 25.2 | -0.024 | 0.019 | 0.209 |
| rs4850842 | G | A | 0.430 | -0.012 | 0.002 | 2.200e-07 | 26.8 | -0.003 | 0.016 | 0.850 |
| rs4856278 | A | G | 0.242 | 0.015 | 0.003 | 1.700e-08 | 31.8 | 0.044 | 0.019 | 0.023 |
| rs4872389 | C | T | 0.770 | -0.012 | 0.003 | 4.700e-06 | 21.0 | -0.006 | 0.018 | 0.743 |
| rs4886868 | T | G | 0.414 | -0.012 | 0.002 | 3.500e-08 | 30.4 | -0.019 | 0.016 | 0.242 |
| rs4906229 | A | C | 0.245 | -0.013 | 0.003 | 5.500e-07 | 25.1 | 0.022 | 0.018 | 0.218 |
| rs527737 | C | T | 0.422 | -0.012 | 0.002 | 6.100e-08 | 29.3 | -0.038 | 0.016 | 0.014 |
| rs559411160 | A | T | 0.582 | 0.014 | 0.002 | 1.100e-08 | 32.7 | -0.007 | 0.022 | 0.770 |
| rs56293069 | G | C | 0.754 | 0.014 | 0.003 | 5.800e-08 | 29.4 | -0.016 | 0.019 | 0.388 |
| rs56947091 | C | T | 0.490 | -0.011 | 0.002 | 2.600e-07 | 26.5 | 0.009 | 0.016 | 0.552 |
| rs59019561 | G | A | 0.951 | -0.025 | 0.005 | 1.600e-06 | 23.1 | 0.024 | 0.038 | 0.521 |
| rs61301535 | C | G | 0.987 | -0.046 | 0.010 | 3.300e-06 | 21.6 | -0.048 | 0.073 | 0.509 |
| rs61780535 | C | T | 0.798 | 0.014 | 0.003 | 3.000e-07 | 26.3 | 0.018 | 0.023 | 0.448 |
| rs61928076 | G | A | 0.955 | -0.024 | 0.005 | 4.400e-06 | 21.1 | 0.019 | 0.057 | 0.738 |
| rs61975407 | C | T | 0.452 | -0.011 | 0.002 | 2.000e-06 | 22.6 | -0.017 | 0.016 | 0.299 |
| rs62252168 | C | T | 0.884 | 0.016 | 0.003 | 4.900e-06 | 20.9 | 0.009 | 0.027 | 0.747 |
| rs62352768 | T | A | 0.955 | -0.028 | 0.006 | 2.600e-06 | 22.1 | 0.106 | 0.079 | 0.177 |
| rs62397687 | C | T | 0.816 | 0.015 | 0.003 | 8.000e-08 | 28.8 | 0.001 | 0.021 | 0.972 |
| rs6427349 | C | A | 0.392 | 0.011 | 0.002 | 2.400e-06 | 22.2 | 0.021 | 0.018 | 0.242 |
| rs6497882 | A | G | 0.404 | 0.011 | 0.002 | 3.300e-06 | 21.6 | 0.014 | 0.016 | 0.377 |
| rs67432617 | A | C | 0.805 | 0.015 | 0.003 | 5.100e-08 | 29.7 | -0.004 | 0.021 | 0.852 |
| rs6743932 | G | A | 0.803 | -0.013 | 0.003 | 4.200e-06 | 21.2 | 0.004 | 0.020 | 0.842 |
| rs6812265 | C | T | 0.548 | 0.011 | 0.002 | 3.300e-07 | 26.1 | -0.005 | 0.016 | 0.751 |
| rs707929 | A | G | 0.665 | 0.012 | 0.002 | 2.300e-07 | 26.7 | 0.006 | 0.016 | 0.714 |
| rs710187 | C | T | 0.442 | 0.010 | 0.002 | 2.200e-06 | 22.4 | 0.003 | 0.016 | 0.843 |
| rs7174255 | G | A | 0.301 | -0.011 | 0.002 | 3.300e-06 | 21.6 | 0.006 | 0.017 | 0.701 |
| rs719263 | T | C | 0.195 | -0.013 | 0.003 | 2.800e-06 | 22.0 | -0.027 | 0.019 | 0.158 |
| rs7233752 | A | G | 0.805 | 0.013 | 0.003 | 3.800e-06 | 21.4 | 0.020 | 0.019 | 0.298 |
| rs72638967 | C | T | 0.981 | -0.038 | 0.008 | 2.300e-06 | 22.3 | 0.017 | 0.117 | 0.885 |
| rs72920838 | C | T | 0.889 | 0.017 | 0.004 | 1.900e-06 | 22.7 | 0.014 | 0.026 | 0.600 |
| rs72928932 | T | G | 0.917 | -0.021 | 0.004 | 2.800e-07 | 26.4 | -0.023 | 0.037 | 0.544 |
| rs7326482 | G | T | 0.385 | -0.013 | 0.002 | 1.600e-08 | 31.9 | -0.001 | 0.016 | 0.963 |
| rs749256 | T | C | 0.250 | 0.013 | 0.003 | 3.800e-07 | 25.8 | 0.044 | 0.021 | 0.038 |
| rs7508365 | T | C | 0.280 | -0.011 | 0.002 | 3.600e-06 | 21.5 | 0.003 | 0.018 | 0.879 |
| rs7652008 | T | C | 0.537 | -0.011 | 0.002 | 1.600e-06 | 23.0 | -0.007 | 0.016 | 0.656 |
| rs7690846 | T | C | 0.484 | 0.010 | 0.002 | 2.700e-06 | 22.0 | 0.015 | 0.017 | 0.372 |
| rs7748167 | A | C | 0.875 | 0.016 | 0.003 | 2.300e-06 | 22.3 | -0.020 | 0.025 | 0.428 |
| rs77742115 | T | C | 0.862 | -0.018 | 0.003 | 9.600e-09 | 32.9 | -0.009 | 0.023 | 0.710 |
| rs7804463 | T | C | 0.530 | 0.015 | 0.002 | 1.200e-11 | 46.0 | 0.004 | 0.016 | 0.819 |
| rs80014012 | A | C | 0.925 | -0.020 | 0.004 | 2.300e-06 | 22.3 | 0.013 | 0.034 | 0.700 |
| rs8058726 | A | T | 0.712 | -0.011 | 0.002 | 2.700e-06 | 22.0 | 0.013 | 0.017 | 0.446 |
| rs862490 | T | C | 0.358 | -0.011 | 0.002 | 2.700e-06 | 22.0 | 0.032 | 0.018 | 0.077 |
| rs877483 | T | C | 0.433 | 0.012 | 0.002 | 4.000e-08 | 30.1 | 0.013 | 0.016 | 0.410 |
| rs887464 | C | T | 0.547 | 0.011 | 0.002 | 4.500e-07 | 25.5 | 0.018 | 0.019 | 0.329 |
| rs909253 | A | G | 0.639 | 0.011 | 0.002 | 1.000e-06 | 23.9 | -0.009 | 0.016 | 0.585 |
| rs921915 | T | C | 0.412 | -0.014 | 0.002 | 5.700e-10 | 38.4 | 0.018 | 0.016 | 0.249 |
| rs9265546 | G | A | 0.777 | 0.015 | 0.003 | 1.200e-06 | 23.6 | -0.022 | 0.026 | 0.406 |
| rs9266639 | C | T | 0.719 | 0.014 | 0.002 | 8.500e-09 | 33.2 | 0.012 | 0.025 | 0.636 |
| rs9271303 | A | G | 0.794 | 0.017 | 0.003 | 6.000e-08 | 29.4 | -0.025 | 0.022 | 0.250 |
| rs9271657 | T | C | 0.784 | 0.017 | 0.003 | 3.000e-09 | 35.2 | -0.012 | 0.025 | 0.622 |
| rs9272278 | C | G | 0.843 | 0.018 | 0.003 | 2.700e-09 | 35.4 | -0.049 | 0.026 | 0.060 |
| rs9274390 | C | T | 0.855 | 0.021 | 0.004 | 5.500e-08 | 29.5 | 0.005 | 0.035 | 0.879 |
| rs9276029 | G | A | 0.841 | 0.016 | 0.003 | 3.600e-07 | 25.9 | 0.014 | 0.032 | 0.666 |
| rs9276758 | G | A | 0.688 | 0.013 | 0.002 | 5.200e-08 | 29.6 | -0.006 | 0.017 | 0.723 |
| rs9316077 | A | C | 0.414 | 0.010 | 0.002 | 4.500e-06 | 21.0 | 0.010 | 0.016 | 0.518 |
| rs9446663 | C | A | 0.704 | 0.013 | 0.002 | 1.600e-07 | 27.5 | -0.005 | 0.018 | 0.763 |
| rs9500927 | G | A | 0.832 | 0.015 | 0.003 | 4.000e-07 | 25.7 | -0.063 | 0.024 | 0.009 |
| rs9513912 | A | G | 0.962 | -0.027 | 0.006 | 4.300e-06 | 21.1 | -0.029 | 0.054 | 0.592 |
| rs9538536 | T | G | 0.299 | -0.011 | 0.002 | 3.400e-06 | 21.6 | 0.000 | 0.017 | 0.988 |
| rs9579775 | A | C | 0.865 | 0.016 | 0.003 | 2.800e-06 | 21.9 | 0.031 | 0.045 | 0.496 |
| rs9584870 | T | C | 0.633 | -0.011 | 0.002 | 2.400e-06 | 22.2 | 0.005 | 0.017 | 0.740 |
| rs974471 | G | A | 0.229 | 0.014 | 0.003 | 2.200e-07 | 26.8 | 0.019 | 0.018 | 0.297 |
| rs9919769 | A | G | 0.774 | -0.012 | 0.003 | 2.500e-06 | 22.1 | -0.007 | 0.018 | 0.684 |
| Self-reported vigorous physical activity | | | | | | | | | | |
| rs10105796 | T | C | 0.740 | 0.007 | 0.001 | 8.100e-07 | 24.3 | 0.021 | 0.017 | 0.214 |
| rs1081105 | A | C | 0.972 | -0.019 | 0.004 | 2.900e-06 | 21.9 | 0.034 | 0.062 | 0.584 |
| rs10828266 | A | G | 0.285 | -0.007 | 0.001 | 3.500e-06 | 21.5 | 0.027 | 0.017 | 0.118 |
| rs10943642 | G | A | 0.791 | 0.008 | 0.002 | 7.100e-07 | 24.6 | -0.027 | 0.021 | 0.186 |
| rs112532934 | A | G | 0.957 | -0.015 | 0.003 | 2.100e-06 | 22.5 | -0.031 | 0.054 | 0.561 |
| rs114254306 | G | A | 0.982 | -0.024 | 0.005 | 2.000e-06 | 22.6 | 0.142 | 0.069 | 0.039 |
| rs115181288 | C | T | 0.945 | -0.013 | 0.003 | 3.300e-06 | 21.6 | 0.053 | 0.043 | 0.218 |
| rs11575919 | A | G | 0.893 | -0.011 | 0.002 | 3.500e-07 | 26.0 | 0.017 | 0.027 | 0.529 |
| rs11643648 | C | T | 0.926 | -0.012 | 0.003 | 1.600e-06 | 23.0 | -0.003 | 0.028 | 0.912 |
| rs11917490 | G | A | 0.275 | 0.007 | 0.001 | 1.500e-06 | 23.1 | 0.026 | 0.017 | 0.114 |
| rs12046141 | G | A | 0.712 | 0.007 | 0.001 | 1.100e-06 | 23.7 | 0.015 | 0.018 | 0.405 |
| rs12480549 | G | A | 0.806 | -0.008 | 0.002 | 3.400e-06 | 21.6 | 0.021 | 0.019 | 0.260 |
| rs1248860 | G | A | 0.484 | -0.010 | 0.001 | 1.100e-13 | 55.3 | -0.006 | 0.016 | 0.680 |
| rs12624919 | T | C | 0.945 | -0.014 | 0.003 | 2.500e-06 | 22.1 | -0.056 | 0.029 | 0.057 |
| rs12692667 | T | G | 0.722 | -0.007 | 0.001 | 3.300e-06 | 21.6 | 0.014 | 0.017 | 0.419 |
| rs12931073 | C | G | 0.875 | -0.009 | 0.002 | 2.400e-06 | 22.2 | 0.003 | 0.025 | 0.900 |
| rs13182417 | G | A | 0.961 | -0.018 | 0.003 | 1.900e-07 | 27.1 | -0.067 | 0.049 | 0.174 |
| rs13212652 | T | G | 0.870 | 0.011 | 0.002 | 2.900e-08 | 30.8 | -0.029 | 0.025 | 0.247 |
| rs13243553 | G | A | 0.608 | 0.009 | 0.001 | 9.000e-11 | 42.0 | 0.015 | 0.016 | 0.339 |
| rs13375273 | G | A | 0.677 | -0.007 | 0.001 | 2.200e-06 | 22.4 | 0.007 | 0.017 | 0.680 |
| rs138557223 | C | T | 0.985 | -0.027 | 0.006 | 1.900e-06 | 22.7 | -0.005 | 0.071 | 0.947 |
| rs140507 | C | T | 0.537 | -0.006 | 0.001 | 4.400e-06 | 21.1 | -0.006 | 0.015 | 0.698 |
| rs141344324 | A | T | 0.990 | 0.032 | 0.007 | 1.700e-06 | 23.0 | -0.220 | 0.766 | 0.773 |
| rs141813169 | C | T | 0.961 | -0.016 | 0.003 | 4.100e-06 | 21.2 | -0.016 | 0.040 | 0.692 |
| rs1461584 | A | G | 0.794 | -0.009 | 0.002 | 8.200e-08 | 28.7 | 0.002 | 0.019 | 0.926 |
| rs147926280 | G | A | 0.969 | 0.018 | 0.004 | 4.100e-06 | 21.2 | -0.056 | 0.069 | 0.418 |
| rs149921656 | G | T | 0.987 | -0.030 | 0.006 | 7.600e-07 | 24.5 | 0.073 | 0.098 | 0.453 |
| rs1573062 | C | T | 0.648 | -0.007 | 0.001 | 1.500e-06 | 23.1 | 0.007 | 0.016 | 0.651 |
| rs169219 | C | A | 0.403 | -0.007 | 0.001 | 2.200e-07 | 26.9 | -0.017 | 0.016 | 0.266 |
| rs17219084 | A | G | 0.624 | -0.006 | 0.001 | 3.200e-06 | 21.7 | -0.035 | 0.018 | 0.047 |
| rs17338135 | C | T | 0.568 | 0.006 | 0.001 | 2.200e-06 | 22.4 | 0.032 | 0.032 | 0.317 |
| rs17597778 | T | G | 0.888 | 0.010 | 0.002 | 3.400e-06 | 21.6 | 0.007 | 0.026 | 0.789 |
| rs1793894 | G | A | 0.832 | 0.009 | 0.002 | 1.400e-07 | 27.7 | -0.021 | 0.040 | 0.605 |
| rs184335918 | T | C | 0.922 | -0.012 | 0.003 | 2.500e-06 | 22.2 | -0.051 | 0.042 | 0.220 |
| rs2050189 | T | C | 0.770 | 0.007 | 0.002 | 4.000e-06 | 21.3 | -0.012 | 0.026 | 0.654 |
| rs2059374 | T | C | 0.182 | 0.010 | 0.002 | 3.900e-09 | 34.6 | -0.007 | 0.021 | 0.741 |
| rs2189464 | C | T | 0.752 | 0.008 | 0.002 | 5.600e-07 | 25.0 | 0.017 | 0.018 | 0.329 |
| rs2326267 | T | C | 0.121 | 0.009 | 0.002 | 3.000e-06 | 21.8 | 0.043 | 0.026 | 0.097 |
| rs2523454 | G | A | 0.658 | 0.007 | 0.001 | 1.500e-06 | 23.1 | -0.040 | 0.026 | 0.126 |
| rs2596496 | C | G | 0.330 | 0.007 | 0.001 | 1.400e-07 | 27.7 | -0.004 | 0.018 | 0.825 |
| rs2663648 | C | T | 0.884 | -0.011 | 0.002 | 2.000e-07 | 27.0 | -0.016 | 0.022 | 0.453 |
| rs2764261 | A | G | 0.374 | 0.009 | 0.001 | 2.000e-11 | 45.0 | 0.003 | 0.016 | 0.867 |
| rs28361032 | A | G | 0.605 | 0.007 | 0.001 | 1.100e-06 | 23.8 | -0.003 | 0.017 | 0.868 |
| rs2844494 | C | A | 0.349 | 0.006 | 0.001 | 3.600e-06 | 21.5 | -0.006 | 0.016 | 0.696 |
| rs288052 | T | A | 0.598 | -0.006 | 0.001 | 3.800e-06 | 21.4 | -0.001 | 0.016 | 0.971 |
| rs2922754 | G | A | 0.242 | 0.007 | 0.002 | 2.200e-06 | 22.4 | 0.024 | 0.018 | 0.187 |
| rs3096698 | T | C | 0.348 | 0.006 | 0.001 | 3.500e-06 | 21.5 | -0.016 | 0.018 | 0.358 |
| rs328902 | C | T | 0.685 | -0.009 | 0.001 | 5.500e-10 | 38.5 | 0.004 | 0.017 | 0.825 |
| rs35907069 | G | A | 0.911 | -0.011 | 0.002 | 1.300e-06 | 23.4 | -0.024 | 0.030 | 0.431 |
| rs3737224 | C | T | 0.901 | 0.010 | 0.002 | 3.500e-06 | 21.5 | 0.022 | 0.027 | 0.410 |
| rs3781411 | C | T | 0.876 | 0.013 | 0.002 | 3.000e-10 | 39.7 | -0.023 | 0.021 | 0.291 |
| rs429358 | T | C | 0.846 | -0.009 | 0.002 | 5.300e-07 | 25.1 | 0.011 | 0.023 | 0.622 |
| rs4556111 | T | C | 0.466 | -0.007 | 0.001 | 2.100e-07 | 26.9 | -0.014 | 0.015 | 0.367 |
| rs464348 | A | G | 0.301 | 0.007 | 0.001 | 1.400e-06 | 23.3 | -0.029 | 0.017 | 0.090 |
| rs469228 | A | G | 0.748 | 0.007 | 0.002 | 4.300e-06 | 21.1 | -0.003 | 0.018 | 0.869 |
| rs4856278 | A | G | 0.242 | 0.010 | 0.002 | 2.000e-10 | 40.5 | 0.044 | 0.019 | 0.023 |
| rs509533 | G | C | 0.539 | 0.007 | 0.001 | 5.800e-07 | 25.0 | -0.014 | 0.016 | 0.362 |
| rs526852 | G | A | 0.422 | -0.007 | 0.001 | 9.400e-07 | 24.0 | -0.038 | 0.016 | 0.014 |
| rs559411160 | A | T | 0.582 | 0.008 | 0.001 | 4.100e-08 | 30.1 | -0.007 | 0.022 | 0.770 |
| rs5742915 | T | C | 0.539 | -0.007 | 0.001 | 5.200e-07 | 25.2 | -0.011 | 0.016 | 0.510 |
| rs577525 | T | C | 0.437 | 0.006 | 0.001 | 2.000e-06 | 22.6 | -0.015 | 0.016 | 0.330 |
| rs58242881 | G | A | 0.793 | 0.009 | 0.002 | 8.600e-08 | 28.7 | -0.016 | 0.021 | 0.440 |
| rs608410 | T | C | 0.275 | 0.007 | 0.001 | 1.200e-06 | 23.5 | -0.016 | 0.017 | 0.353 |
| rs61866271 | G | C | 0.983 | 0.028 | 0.005 | 6.900e-08 | 29.1 | 0.081 | 0.075 | 0.276 |
| rs61897072 | C | T | 0.805 | 0.009 | 0.002 | 2.700e-07 | 26.5 | -0.010 | 0.020 | 0.626 |
| rs62097177 | A | T | 0.970 | 0.019 | 0.004 | 2.900e-06 | 21.9 | 0.024 | 0.055 | 0.664 |
| rs62516465 | C | T | 0.744 | -0.007 | 0.002 | 4.500e-06 | 21.0 | -0.005 | 0.018 | 0.769 |
| rs6432141 | A | G | 0.446 | 0.006 | 0.001 | 2.200e-06 | 22.4 | 0.007 | 0.016 | 0.665 |
| rs6562062 | G | A | 0.372 | -0.006 | 0.001 | 3.200e-06 | 21.7 | 0.012 | 0.016 | 0.451 |
| rs6667222 | A | C | 0.748 | 0.009 | 0.002 | 8.700e-09 | 33.1 | 0.025 | 0.018 | 0.164 |
| rs6689056 | G | A | 0.674 | 0.008 | 0.001 | 6.500e-08 | 29.2 | -0.010 | 0.018 | 0.591 |
| rs67306275 | T | C | 0.857 | 0.009 | 0.002 | 4.800e-06 | 20.9 | -0.002 | 0.023 | 0.938 |
| rs67432617 | A | C | 0.806 | 0.008 | 0.002 | 3.800e-06 | 21.4 | -0.004 | 0.021 | 0.852 |
| rs72714728 | G | A | 0.993 | -0.036 | 0.008 | 2.900e-06 | 21.9 | 0.286 | 0.121 | 0.018 |
| rs72737787 | G | A | 0.648 | -0.006 | 0.001 | 2.400e-06 | 22.2 | 0.020 | 0.016 | 0.231 |
| rs72924609 | G | A | 0.875 | 0.010 | 0.002 | 1.500e-07 | 27.6 | 0.012 | 0.025 | 0.637 |
| rs72928932 | T | G | 0.918 | -0.013 | 0.002 | 7.500e-08 | 28.9 | -0.023 | 0.037 | 0.544 |
| rs72993373 | T | C | 0.971 | -0.019 | 0.004 | 1.200e-06 | 23.6 | 0.043 | 0.045 | 0.331 |
| rs73074777 | A | G | 0.664 | 0.007 | 0.001 | 2.700e-06 | 22.0 | 0.007 | 0.017 | 0.687 |
| rs73132101 | T | G | 0.851 | 0.009 | 0.002 | 7.200e-07 | 24.6 | -0.004 | 0.022 | 0.851 |
| rs73141532 | T | G | 0.548 | 0.006 | 0.001 | 2.500e-06 | 22.1 | 0.015 | 0.017 | 0.365 |
| rs73912051 | T | C | 0.937 | 0.012 | 0.003 | 4.800e-06 | 20.9 | 0.046 | 0.035 | 0.191 |
| rs74427596 | G | C | 0.954 | -0.015 | 0.003 | 2.000e-06 | 22.6 | 0.032 | 0.040 | 0.435 |
| rs75466549 | C | G | 0.861 | 0.009 | 0.002 | 4.600e-06 | 21.0 | -0.045 | 0.028 | 0.111 |
| rs7715147 | C | A | 0.758 | -0.007 | 0.002 | 4.600e-06 | 21.0 | 0.001 | 0.025 | 0.975 |
| rs7749938 | C | T | 0.985 | 0.026 | 0.006 | 2.800e-06 | 22.0 | 0.050 | 0.060 | 0.403 |
| rs77742115 | T | C | 0.862 | -0.010 | 0.002 | 5.900e-07 | 24.9 | -0.009 | 0.023 | 0.710 |
| rs78651718 | G | A | 0.975 | -0.020 | 0.004 | 4.800e-06 | 20.9 | 0.016 | 0.048 | 0.732 |
| rs80292109 | A | G | 0.724 | -0.008 | 0.001 | 3.900e-07 | 25.8 | 0.027 | 0.017 | 0.110 |
| rs8102851 | T | C | 0.783 | 0.008 | 0.002 | 1.900e-06 | 22.7 | -0.019 | 0.019 | 0.309 |
| rs9266639 | C | T | 0.719 | 0.007 | 0.001 | 5.700e-07 | 25.0 | 0.012 | 0.025 | 0.636 |
| rs9267653 | T | C | 0.287 | 0.007 | 0.001 | 2.300e-06 | 22.4 | -0.011 | 0.017 | 0.539 |
| rs9270861 | G | A | 0.732 | 0.007 | 0.002 | 1.300e-06 | 23.5 | 0.007 | 0.023 | 0.767 |
| rs9271112 | T | C | 0.291 | 0.007 | 0.001 | 1.700e-06 | 22.9 | 0.033 | 0.026 | 0.199 |
| rs9271303 | A | G | 0.794 | 0.009 | 0.002 | 1.100e-06 | 23.7 | -0.025 | 0.022 | 0.250 |
| rs9271364 | A | G | 0.496 | 0.007 | 0.001 | 3.200e-08 | 30.6 | -0.037 | 0.022 | 0.094 |
| rs9273051 | G | A | 0.750 | 0.008 | 0.002 | 1.100e-07 | 28.1 | -0.067 | 0.026 | 0.009 |
| rs9275602 | C | A | 0.803 | 0.008 | 0.002 | 2.800e-06 | 21.9 | -0.045 | 0.037 | 0.217 |
| rs9276758 | G | A | 0.688 | 0.008 | 0.001 | 1.400e-08 | 32.1 | -0.006 | 0.017 | 0.723 |
| rs9384757 | G | A | 0.790 | -0.008 | 0.002 | 1.700e-06 | 23.0 | -0.027 | 0.019 | 0.166 |
| rs9387216 | C | A | 0.748 | -0.008 | 0.002 | 2.000e-07 | 27.1 | -0.021 | 0.017 | 0.219 |
| rs974471 | G | A | 0.228 | 0.007 | 0.002 | 4.200e-06 | 21.2 | 0.019 | 0.018 | 0.297 |
| rs9984123 | T | G | 0.340 | -0.007 | 0.001 | 1.600e-06 | 23.0 | -0.010 | 0.019 | 0.608 |
| Accelerometery - average acceleration | | | | | | | | | | |
| rs10120942 | C | A | 0.430 | 0.172 | 0.037 | 2.600e-06 | 22.1 | 0.007 | 0.016 | 0.630 |
| rs10415609 | A | G | 0.122 | 0.265 | 0.055 | 1.700e-06 | 22.9 | 0.012 | 0.024 | 0.607 |
| rs10475334 | G | A | 0.872 | 0.249 | 0.054 | 4.500e-06 | 21.0 | 0.048 | 0.025 | 0.055 |
| rs10809491 | T | C | 0.745 | 0.201 | 0.042 | 1.400e-06 | 23.2 | 0.029 | 0.018 | 0.104 |
| rs10880697 | G | C | 0.661 | -0.204 | 0.038 | 1.200e-07 | 28.0 | -0.010 | 0.018 | 0.590 |
| rs10912763 | C | T | 0.756 | 0.202 | 0.042 | 1.700e-06 | 22.9 | -0.013 | 0.018 | 0.457 |
| rs11007336 | G | A | 0.537 | 0.173 | 0.037 | 2.600e-06 | 22.1 | -0.001 | 0.016 | 0.971 |
| rs11012732 | A | G | 0.668 | 0.225 | 0.039 | 5.400e-09 | 34.0 | -0.024 | 0.017 | 0.154 |
| rs111789382 | T | C | 0.934 | -0.361 | 0.076 | 1.800e-06 | 22.8 | -0.028 | 0.038 | 0.465 |
| rs112779734 | T | C | 0.692 | 0.190 | 0.039 | 1.400e-06 | 23.3 | 0.004 | 0.017 | 0.792 |
| rs11548275 | C | T | 0.975 | -0.537 | 0.116 | 3.800e-06 | 21.4 | 0.060 | 0.073 | 0.414 |
| rs11865683 | C | G | 0.528 | -0.183 | 0.036 | 4.400e-07 | 25.5 | -0.009 | 0.016 | 0.565 |
| rs12045968 | T | G | 0.783 | -0.239 | 0.044 | 5.100e-08 | 29.7 | -0.023 | 0.019 | 0.223 |
| rs1220114 | T | A | 0.743 | 0.194 | 0.042 | 4.800e-06 | 20.9 | -0.022 | 0.018 | 0.216 |
| rs12446623 | T | G | 0.855 | -0.245 | 0.052 | 2.200e-06 | 22.4 | -0.028 | 0.020 | 0.163 |
| rs12448218 | G | A | 0.661 | -0.188 | 0.038 | 1.000e-06 | 23.9 | -0.016 | 0.017 | 0.336 |
| rs12460611 | A | G | 0.677 | 0.188 | 0.039 | 1.300e-06 | 23.4 | -0.021 | 0.017 | 0.218 |
| rs12522261 | G | A | 0.657 | 0.211 | 0.038 | 3.900e-08 | 30.2 | -0.006 | 0.016 | 0.728 |
| rs1268539 | C | A | 0.581 | -0.189 | 0.037 | 2.700e-07 | 26.4 | 0.037 | 0.016 | 0.019 |
| rs139552414 | C | T | 0.977 | -0.555 | 0.120 | 3.700e-06 | 21.4 | 0.049 | 0.058 | 0.401 |
| rs144277946 | C | T | 0.977 | 0.566 | 0.123 | 4.500e-06 | 21.0 | -0.048 | 0.063 | 0.449 |
| rs144567362 | T | G | 0.276 | -0.209 | 0.045 | 3.900e-06 | 21.3 | 0.029 | 0.032 | 0.360 |
| rs1465406 | A | C | 0.771 | -0.200 | 0.043 | 4.200e-06 | 21.2 | -0.011 | 0.019 | 0.554 |
| rs147350938 | C | T | 0.987 | -0.785 | 0.165 | 2.000e-06 | 22.6 | 0.130 | 0.092 | 0.158 |
| rs148193266 | A | C | 0.957 | -0.510 | 0.092 | 3.100e-08 | 30.7 | 0.001 | 0.041 | 0.986 |
| rs154103 | T | C | 0.344 | -0.188 | 0.038 | 1.000e-06 | 23.9 | -0.015 | 0.016 | 0.368 |
| rs1550435 | T | C | 0.571 | 0.200 | 0.037 | 5.000e-08 | 29.7 | -0.011 | 0.016 | 0.479 |
| rs1550437 | C | T | 0.832 | 0.229 | 0.048 | 2.300e-06 | 22.3 | 0.000 | 0.020 | 0.999 |
| rs157595 | A | G | 0.385 | -0.180 | 0.038 | 2.400e-06 | 22.2 | 0.018 | 0.018 | 0.297 |
| rs159963 | C | A | 0.422 | 0.172 | 0.037 | 3.100e-06 | 21.8 | -0.030 | 0.016 | 0.055 |
| rs1668835 | T | A | 0.688 | -0.198 | 0.039 | 4.800e-07 | 25.4 | 0.012 | 0.017 | 0.492 |
| rs16860042 | T | C | 0.904 | -0.288 | 0.062 | 3.100e-06 | 21.8 | -0.004 | 0.029 | 0.881 |
| rs17135735 | C | T | 0.929 | 0.340 | 0.071 | 1.600e-06 | 23.1 | 0.019 | 0.034 | 0.582 |
| rs17203754 | G | A | 0.891 | -0.275 | 0.059 | 3.200e-06 | 21.7 | -0.058 | 0.028 | 0.038 |
| rs17692129 | C | T | 0.665 | 0.191 | 0.038 | 6.700e-07 | 24.7 | 0.045 | 0.026 | 0.085 |
| rs2095618 | C | T | 0.711 | -0.185 | 0.040 | 4.200e-06 | 21.2 | -0.003 | 0.017 | 0.858 |
| rs34517439 | C | A | 0.879 | 0.308 | 0.056 | 4.400e-08 | 30.0 | 0.018 | 0.030 | 0.550 |
| rs4040769 | G | A | 0.637 | -0.178 | 0.038 | 2.400e-06 | 22.2 | 0.013 | 0.016 | 0.402 |
| rs4621706 | C | T | 0.457 | 0.169 | 0.037 | 3.700e-06 | 21.4 | 0.006 | 0.016 | 0.684 |
| rs4640189 | C | T | 0.934 | -0.351 | 0.073 | 1.800e-06 | 22.8 | -0.015 | 0.042 | 0.717 |
| rs4779063 | A | G | 0.223 | -0.220 | 0.044 | 4.300e-07 | 25.5 | -0.033 | 0.020 | 0.092 |
| rs4906247 | C | A | 0.350 | 0.175 | 0.038 | 4.500e-06 | 21.0 | -0.007 | 0.017 | 0.695 |
| rs4921269 | A | C | 0.390 | -0.175 | 0.037 | 2.700e-06 | 22.0 | 0.009 | 0.016 | 0.551 |
| rs55938136 | A | G | 0.776 | -0.292 | 0.044 | 2.100e-11 | 44.9 | -0.078 | 0.039 | 0.046 |
| rs56194509 | T | G | 0.780 | -0.303 | 0.044 | 5.000e-12 | 47.7 | -0.009 | 0.029 | 0.758 |
| rs59499656 | A | T | 0.656 | -0.228 | 0.038 | 2.400e-09 | 35.6 | -0.004 | 0.016 | 0.820 |
| rs6081105 | A | G | 0.399 | -0.183 | 0.037 | 8.200e-07 | 24.3 | -0.015 | 0.018 | 0.394 |
| rs62045887 | T | C | 0.753 | -0.194 | 0.042 | 4.000e-06 | 21.3 | -0.013 | 0.018 | 0.486 |
| rs62125156 | A | C | 0.788 | 0.204 | 0.045 | 4.500e-06 | 21.0 | 0.042 | 0.021 | 0.046 |
| rs62168614 | G | A | 0.708 | 0.198 | 0.040 | 6.000e-07 | 24.9 | -0.019 | 0.018 | 0.295 |
| rs6540895 | T | C | 0.572 | 0.170 | 0.037 | 3.400e-06 | 21.6 | 0.008 | 0.016 | 0.635 |
| rs6541235 | C | T | 0.852 | 0.252 | 0.051 | 7.300e-07 | 24.5 | 0.006 | 0.023 | 0.784 |
| rs6775319 | A | T | 0.271 | 0.225 | 0.041 | 3.500e-08 | 30.4 | -0.029 | 0.017 | 0.095 |
| rs72804364 | G | A | 0.823 | 0.255 | 0.048 | 9.700e-08 | 28.4 | 0.004 | 0.020 | 0.834 |
| rs73249344 | A | G | 0.814 | -0.219 | 0.047 | 2.900e-06 | 21.9 | -0.025 | 0.022 | 0.259 |
| rs7557793 | T | C | 0.779 | -0.201 | 0.044 | 4.300e-06 | 21.1 | 0.013 | 0.018 | 0.487 |
| rs7591144 | G | A | 0.613 | -0.173 | 0.037 | 3.200e-06 | 21.7 | 0.021 | 0.016 | 0.180 |
| rs75972257 | C | A | 0.912 | 0.313 | 0.065 | 1.400e-06 | 23.3 | 0.040 | 0.036 | 0.266 |
| rs7626095 | C | T | 0.562 | -0.176 | 0.037 | 2.200e-06 | 22.4 | -0.014 | 0.018 | 0.414 |
| rs7658462 | C | T | 0.828 | 0.234 | 0.048 | 1.200e-06 | 23.6 | 0.012 | 0.020 | 0.537 |
| rs7841628 | C | T | 0.888 | -0.264 | 0.058 | 4.600e-06 | 21.0 | -0.027 | 0.024 | 0.256 |
| rs78982639 | G | A | 0.989 | 0.882 | 0.172 | 2.800e-07 | 26.4 | 0.049 | 0.108 | 0.648 |
| rs791273 | A | G | 0.748 | -0.222 | 0.042 | 1.100e-07 | 28.2 | -0.002 | 0.017 | 0.907 |
| rs8100652 | G | C | 0.683 | -0.184 | 0.039 | 2.300e-06 | 22.3 | -0.005 | 0.016 | 0.753 |
| rs9293503 | T | C | 0.888 | 0.329 | 0.059 | 2.100e-08 | 31.4 | -0.007 | 0.025 | 0.778 |
| rs9300002 | T | C | 0.579 | -0.175 | 0.037 | 2.100e-06 | 22.5 | 0.001 | 0.016 | 0.941 |
| rs945890 | A | T | 0.287 | -0.209 | 0.040 | 2.200e-07 | 26.9 | -0.029 | 0.017 | 0.092 |
| rs9529057 | G | A | 0.526 | -0.174 | 0.036 | 1.700e-06 | 22.9 | -0.016 | 0.016 | 0.335 |
| rs9938281 | A | G | 0.474 | -0.192 | 0.037 | 1.500e-07 | 27.7 | -0.027 | 0.016 | 0.087 |
| Accelerometery – fraction of time with acceleration 425 milli-gravities | | | | | | | | | | |
| rs10465810 | A | T | 0.607 | 0.020 | 0.004 | 2.800e-06 | 21.9 | 0.013 | 0.016 | 0.433 |
| rs11866559 | G | A | 0.820 | -0.027 | 0.005 | 7.200e-07 | 24.6 | 0.021 | 0.019 | 0.270 |
| rs1358994 | G | C | 0.663 | -0.022 | 0.005 | 3.000e-06 | 21.8 | 0.017 | 0.018 | 0.346 |
| rs1454556 | T | C | 0.626 | -0.020 | 0.004 | 1.600e-06 | 23.1 | -0.008 | 0.016 | 0.617 |
| rs1668835 | T | A | 0.688 | -0.023 | 0.004 | 3.100e-07 | 26.2 | 0.012 | 0.017 | 0.492 |
| rs17221323 | T | C | 0.812 | -0.024 | 0.005 | 4.900e-06 | 20.9 | 0.004 | 0.029 | 0.881 |
| rs17395021 | T | C | 0.871 | -0.029 | 0.006 | 2.000e-06 | 22.6 | 0.007 | 0.025 | 0.786 |
| rs1856329 | A | C | 0.801 | 0.027 | 0.005 | 9.000e-08 | 28.6 | -0.015 | 0.019 | 0.433 |
| rs2867655 | C | G | 0.161 | 0.027 | 0.006 | 1.500e-06 | 23.1 | -0.033 | 0.022 | 0.140 |
| rs4314553 | T | C | 0.516 | 0.021 | 0.004 | 2.500e-07 | 26.6 | 0.001 | 0.016 | 0.957 |
| rs4419891 | T | C | 0.278 | 0.022 | 0.005 | 2.400e-06 | 22.3 | 0.007 | 0.017 | 0.667 |
| rs4754194 | C | T | 0.773 | -0.025 | 0.005 | 2.400e-07 | 26.6 | 0.019 | 0.018 | 0.298 |
| rs55938136 | A | G | 0.776 | -0.024 | 0.005 | 8.400e-07 | 24.3 | -0.078 | 0.039 | 0.046 |
| rs61276363 | G | T | 0.808 | -0.025 | 0.005 | 3.000e-06 | 21.8 | -0.038 | 0.021 | 0.064 |
| rs62125156 | A | C | 0.788 | 0.024 | 0.005 | 1.900e-06 | 22.7 | 0.042 | 0.021 | 0.046 |
| rs62443625 | T | C | 0.767 | -0.026 | 0.005 | 1.400e-07 | 27.7 | 0.011 | 0.020 | 0.575 |
| rs62460842 | G | A | 0.920 | 0.035 | 0.008 | 4.000e-06 | 21.3 | 0.006 | 0.030 | 0.851 |
| rs6433478 | T | C | 0.457 | -0.024 | 0.004 | 1.200e-08 | 32.5 | 0.007 | 0.016 | 0.667 |
| rs6579971 | A | G | 0.292 | 0.021 | 0.005 | 3.000e-06 | 21.8 | 0.005 | 0.017 | 0.752 |
| rs6754234 | C | T | 0.097 | 0.032 | 0.007 | 3.400e-06 | 21.6 | -0.010 | 0.024 | 0.695 |
| rs72633364 | G | A | 0.711 | -0.023 | 0.005 | 4.100e-07 | 25.6 | 0.003 | 0.018 | 0.844 |
| rs743580 | A | G | 0.510 | 0.025 | 0.004 | 1.300e-09 | 36.8 | -0.004 | 0.015 | 0.781 |
| rs74863845 | C | G | 0.977 | -0.066 | 0.014 | 2.500e-06 | 22.2 | 0.077 | 0.056 | 0.170 |
| rs7547571 | G | T | 0.424 | 0.020 | 0.004 | 1.500e-06 | 23.2 | 0.001 | 0.016 | 0.935 |
| rs77598509 | G | A | 0.975 | 0.061 | 0.013 | 3.600e-06 | 21.5 | 0.035 | 0.069 | 0.613 |
| rs77981032 | G | A | 0.953 | 0.046 | 0.010 | 3.400e-06 | 21.6 | -0.005 | 0.041 | 0.902 |
| rs78830914 | A | G | 0.971 | -0.059 | 0.012 | 1.600e-06 | 23.0 | -0.031 | 0.062 | 0.614 |
| rs80028338 | A | C | 0.795 | -0.028 | 0.005 | 1.500e-07 | 27.6 | -0.009 | 0.029 | 0.752 |

SNP, single nucleotide polymorphism. EA, effect allele. OA, other allele. EAF, effect allele frequency. SE, standard error.

Supplementary Table 2 Heterogeneity of Wald ratios and MR-Egger test for directional pleiotropy

| Exposure | Heterogeneity | | |  |
| --- | --- | --- | --- | --- |
|  | Q | Degrees of Freedom | P | I² |
| Self-reported moderate-to-vigorous physical activity | 136.15 | 152 | 8.170e-01 | 0.001 |
| Self-reported vigorous physical activity | 109.18 | 101 | 2.719e-01 | 0.067 |
| Accelerometery - average acceleration | 73.42 | 67 | 2.759e-01 | 0.077 |
| Accelerometery – fraction of time with acceleration 425 milli-gravities | 22.24 | 27 | 7.250e-01 | 0.001 |
|  | MR-Egger test for directional pleiotropy | | |  |
|  | Intercept | Standard error | P |  |
| Self-reported moderate-to-vigorous physical activity | 1.111e-02 | 0.007 | 9.827e-02 |  |
| Self-reported vigorous physical activity | 3.081e-03 | 0.008 | 6.923e-01 |  |
| Accelerometery - average acceleration | -5.546e-03 | 0.011 | 6.043e-01 |  |
| Accelerometery – fraction of time with acceleration 425 milli-gravities | 1.568e-02 | 0.017 | 3.727e-01 |  |
